# ERK Signaling Controls Innate-like CD8^+^ T Cell Differentiation via the ELK4 (SAP-1) and ELK1 Transcription Factors

**DOI:** 10.4049/jimmunol.1800704

**Published:** 2018-08-01

**Authors:** Diane Maurice, Patrick Costello, Mathew Sargent, Richard Treisman

**Affiliations:** Signalling and Transcription Group, The Francis Crick Institute, London NW1 1AT, United Kingdom

## Abstract

In mouse thymocyte development, signaling by the TCR through the ERK pathway is required for positive selection of conventional naive T cells. The Ets transcription factor ELK4 (SAP-1), an ERK-regulated cofactor of the SRF transcription factor, plays an important role in positive selection by activating immediate-early genes such as the Egr transcription factor family. The role of ELK4–SRF signaling in development of other T cell types dependent on ERK signaling has been unclear. In this article, we show that ELK4, and its close relative ELK1, act cell autonomously in the thymus to control the generation of innate-like αβ CD8^+^ T cells with memory-like characteristics. Mice lacking ELK4 and ELK1 develop increased numbers of innate-like αβ CD8^+^ T cells, which populate the periphery. These cells develop cell autonomously rather than through expansion of PLZF^+^ thymocytes and concomitantly increased IL-4 signaling. Their development is associated with reduced TCR-mediated activation of ELK4–SRF target genes and can be partially suppressed by overexpression of the ELK4–SRF target gene EGR2. Consistent with this, partial inhibition of ERK signaling in peripheral CD8^+^T cells promotes the generation of cells with innate-like characteristics. These data establish that low-level ERK signaling through ELK4 (and ELK1) promotes innate-like αβ CD8^+^ T cell differentiation, tuning conventional versus innate-like development.

## Introduction

During development of conventional αβ T cells in the thymus, weak TCR signals ensure survival of non–self-reactive thymocytes, whereas strong TCR signaling in self-reactive thymocytes drives their apoptotic elimination (reviewed by Ref. 1, 2). ERK signaling downstream of TCR engagement is essential for thymocyte positive selection but not for negative selection (3, 4). TCR signaling is also important for development of innate-like αβ CD8^+^ T cells, which express high levels of the Eomes transcription factor and which manifest effector functions immediately upon challenge (5–7). For example, *Itk*-null and *Slp76^Y145F^* mutations impair positive selection but increase innate-like αβ CD8^+^ T cell numbers (8–11). At least in the case of Itk, these phenotypes reflect diminished ERK signaling (8, 9), suggesting that weak ERK signaling from lower-affinity TCRs favors innate-like T cell development (reviewed by Ref. 6, 7).

The study of innate αβ CD8^+^ T cell development is complicated because it can occur both cell autonomously and in response to cell-extrinsic cues. The latter includes IL-4, which is produced by cells expressing the PLZF transcription factor and influenced by the *Itk*, *Klf2*, *Id3*, *Irf4*, and *Cbp* genes, and lymphopenic conditions in the periphery (12, 13; for review, see Ref. 14). Nevertheless, the *Nfkb1* and *Bcl11* genes contribute cell autonomously to development of innate-like CD8+ T cells, whereas the effects of *Itk* and *Irf4* are at least partly cell autonomous (15–17). *Irf4* is directly induced in response to TCR signaling in an Itk-dependent manner (17), but the relation of *Nfkb1* and *Bcl11* to TCR signaling remains to be elucidated.

The Ets domain transcription factors SAP-1/*Elk4* and Elk-1/*Elk1* are important nuclear effectors of TCR-induced ERK signaling, acting redundantly in partnership with their DNA-targeting partner SRF (for review, see Ref. 18). Like the ERKs, ELK4/ELK1–SRF signaling is required for positive but not negative selection (19–22). Consistent with this, ELK4/ELK1–SRF targets such as the *Egr1*, *Egr2*, *Egr3*, and *Id3* all promote positive selection (23–26). These data are consistent with a model in which the efficiency of positive selection reflects the strength of ERK signaling to these genes (19, 20).

Given the relationship between TCR signal strength and innate-like αβ CD8^+^ T cell development, we set out to evaluate the contribution of ELK4 and ELK1. We demonstrate that ERK signaling to ELK4 and ELK1 acts to limit differentiation of innate-like αβ CD8^+^ T cells in the thymus and periphery, at least in part through expression of the ELK4–SRF target *Egr2*. Dampening of the ERK signaling upstream of these factors similarly generates peripheral CD8^+^ T cells with memory-like characteristics. Thus, in response to TCR signals, the ELK4–SRF pathway is central to directing differentiation programs in αβ CD8^+^ T cells.

## Materials and Methods

### Animals and reagents

Mice were *Elk4^−/−^* and *Elk4^−/−^ Elk1^−/−^* (19, 20), carrying CD45.1 or CD45.2 alloantigen markers and the F5 TCR transgene (with *Rag2*-null background as specified in the text), and Egr2Tg and CD4Cre deleter (25). Young adult (6–10 wk) age- and sex-matched animals were used in all experiments. For reconstitution, 8-to-10-wk-old female *Rag2*^−/−^ mice watered on acidified water for 1 wk were irradiated (twice with 500 rad separated by 3-h interval), and bone marrow from the femurs of 6-to-8-wk-old donor mice (0.5–1.0 × 10^6^ cells) was injected into the tail vein. Analysis was performed 6 wk later. In some experiments, bone marrow was depleted of T cells and experimental (CD45.2^+^) cells were mixed with (CD45.1^+^CD45.2^+^) cells, and 5 × 10^6^ cells were injected into lethally irradiated B6 SJL (CD45.1^+^) hosts, having received two 600-rad doses at a 3-h interval. Animals were maintained under specific-pathogen–free conditions in the Crick Biological Resources Unit. Animal experimentation was approved by the Crick Animal Ethics committee and carried out under Home Office Procedure Project License 80/2602.

### Flow cytometry

For cell surface staining, cell suspensions were labeled with Abs in PBS, 1% FCS, 2 mM EDTA. For intracellular staining, cells were fixed and permeabilized using Foxp3 staining buffer kit (BD Biosciences) or the BD cytofix/cytoperm kit. For pERK staining, cells were fixed in 2% paraformaldehyde, washed and permeabilized in ice-cold methanol for 30 min, washed twice in PBS 10% FCS, and stained for 1 h. Samples were run on LSR-IIB or Fortessa II (BD Biosciences) and analyzed with FlowJo software (Free Star). An Aria III cell sorter (BD Biosciences) was used to isolate naive CD8 T cells at >97% purity as judged by cell surface marker expression. Coulter CC Size standard beads (Beckman Coulter) were used for calculating cell numbers. Abs were from eBioscience: CD8 (53-6.7), CD44 (IM7), CD122 (5H4), CXCR3 (173), CCR7 (4B12), NKG2D (CX5), CD45.1 (A20), Eomes (Dan11mag), T-bet (4B10), IL-4 (11B11), IFN-γ (XMG1.2), TNF-α (MP6-XT22), and GranzymeB (NGZB); BD Biosciences: Bcl-2 (3F11), CD4 (RM4-5), αβ TCR (β-chain. H57-597), heat stable Ag (HSA; M1/69), and pan ERK; BioLegend: CCL5 (2E9), PLZF (9E12), and IL-4R (I015F8); Santa Cruz Biotechnology: Egr1 (C19); and Cell Signaling Technology: pERK (p42/p44). mCD1d/PBS57 tetramers were generously supplied by the National Institute of Allergy and Infectious Disease MHC Tetramer Core Facility.

### Cell differentiation and stimulation

Sorted naive αβ CD8^+^ T cells were cultured for 2 h in RPMI 1640 containing 10% FCS, 50 μM 2-ME, and penicillin and streptomycin antibiotics (Sigma-Aldrich). For stimulation, 1 × 10^6^ cells were spun (1500 rpm for 5 min) onto αCD3-coated (10 μg/ml) and αCD28-coated (10 μg/ml) 48-well plates washed in PBS and incubated at 37°C 10% CO_2_ before analysis. For CD8^+^ T effector differentiation cultures, cells were washed three times after stimulation and then cultured with 20 ng/ml recombinant IL-2 (ImmunoTools). For fetal thymic organ culture (FTOC), E15.5 fetal thymic lobes were cultured on Medicell membrane floated on IMDM/10% FCS, then disaggregated in accutase (PAA Laboratories) before FACS analysis. For intracellular cytokine staining, cells were stimulated with phorbol 12,13-dibutyrate (PDBu; 50 ng/ml) and ionomycin (1 μg/ml) (both Sigma-Aldrich) in the presence of Brefeldin A (5 μg/ml) (Sigma-Aldrich) for 5 h at 37°C before staining. For IL-4 intracellular staining, thymocyte suspensions were cultured for 1 h with 1.5 μM ionomycin and 50 ng/ml PMA (Sigma-Aldrich), then cultured for 2 h with the addition of Brefeldin A (5 μg/ml).

### Cell proliferation assays

To assess cell division kinetics, FACS-sorted αβ CD8^+^ T cells were stained with CFSE (5 μM; Molecular Probes) for 15 min in RPMI 1640 without serum, washed in 10% serum RPMI 1640 to stop the reaction, and subsequently plated with anti-CD3 and CD28 or NP68 peptide.

### Real-time PCR

Total RNA was extracted from sorted purified αβ CD8^+^ T cells using a GenElute mammalian total RNA kit (Sigma-Aldrich) and subjected to DNase I treatment (Ambion), and cDNA was generated using the Transcriptor cDNA synthetic kit (Roche). Target genes were analyzed using SYBR Green–based real-time PCR (Invitrogen, Carlsbad, CA). Relative template cDNA abundance was calculated using standard curve method, normalizing to Rps16 cDNA, whose level was invariant under all conditions. Primers sequences are available upon request.

### Statistical analysis

Data were analyzed with Graph Pad Prism 6. Bar and dot charts are expressed as mean ± SEM, and data were analyzed using the unpaired and paired parametric *t* test.

## Results

### ELK4 and ELK1 inactivation increases numbers of thymic innate-like CD8^+^ T cells

**FIGURE 1.**
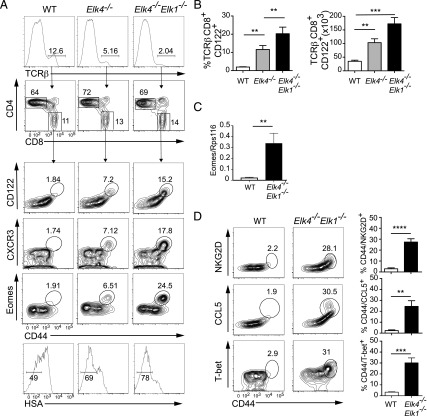
Inactivation of *Elk4* and *Elk1* increases numbers of thymic innate-like αβ CD8^+^ T cells. (**A**) Top panels, TCRβ staining in thymocytes isolated from 8-to-12-wk-old WT, *Elk4^−/−^*, and *Elk4^−/−^Elk-1^−/−^* female animals, with proportions of CD4 and CD8 in TCRβ^hi^-gated thymocytes below. Lower panels, TCRβ^hi^ CD8^+^-gated thymocytes were stained for cell surface expression of CD44, CD122, CXCR3, HSA, and intracellular Eomes. Gated percentages are indicated. (**B**) Proportions (left) and absolute cell numbers (right) of TCRβ^hi^ CD8^+^ CD122^+^ innate T cells in WT, *Elk4^−/−^*, and *Elk4^−/−^ Elk1^−/−^* thymus. Data are representative of three independent staining experiments with ≥5 animals per genotype. (**C**) Levels of Eomes mRNA transcripts in WT and *Elk4^−/−^Elk1^−/−^* purified CD8^+^ SP thymocytes, three animals per genotype. Data are representative of three independent experiments. (**D**) TCRβ^hi^ CD8^+^-gated WT and *Elk4^−/−^ Elk1^−/−^* thymocytes were stained for cell surface expression of NKG2D, intracellular CCL5, and T-bet. Proportions of TCRβ^hi^ CD8^+^ CD44^+^ NKG2D^+^, CCL5^+^, or T-bet^+^ cells are shown. *n* = 5 animals for each genotype; error bars represent SEM. Statistical significance: **p < 0.01, ***p < 0.001, ****p < 0.0001 (unpaired *t* test).

We investigated thymic innate-like T cell development in animals carrying previously characterized mutations in the SRF cofactors SAP-1/*Elk4* and Elk-1/*Elk1* (19, 20). As previously reported, *Elk4*-null animals had reduced αβ TCR^hi^ single-positive (SP) frequency while maintaining the CD4:CD8 ratio, and this was enhanced by *Elk1* inactivation [Fig. 1A (20)]. However, analysis of mature *Elk4*-null CD8^+^ TCRβ^high^ thymocytes revealed an increased proportion and absolute number of cells expressing markers associated with innate-like T cells, including the cell surface memory markers CD44 and CD122 (IL2Rβ) (Fig. 1A, 1B). Increased frequencies of cells expressing the inflammatory chemokine receptor CXCR3 and the memory cell transcription factor eomesodermin (Eomes) (27, 28) were also observed, and Eomes transcript levels were also increased (Fig. 1A, 1C). *Elk4*-null αβ CD8^+^ SP thymocytes appeared more mature than wild-type (WT) cells, exhibiting lower levels of HSA (CD24) (Fig. 1A). All these phenotypes were exacerbated by additional deletion of Elk-1 (Fig. 1A). The *Elk4 Elk1*–null innate-like αβ CD8^+^ SP thymocyte population expressed markers associated with conventional memory cells but not IL-4–induced memory-like cells, such as NKG2D, T-bet, and CCL5 (29) (Fig. 1D).

**FIGURE 2.**
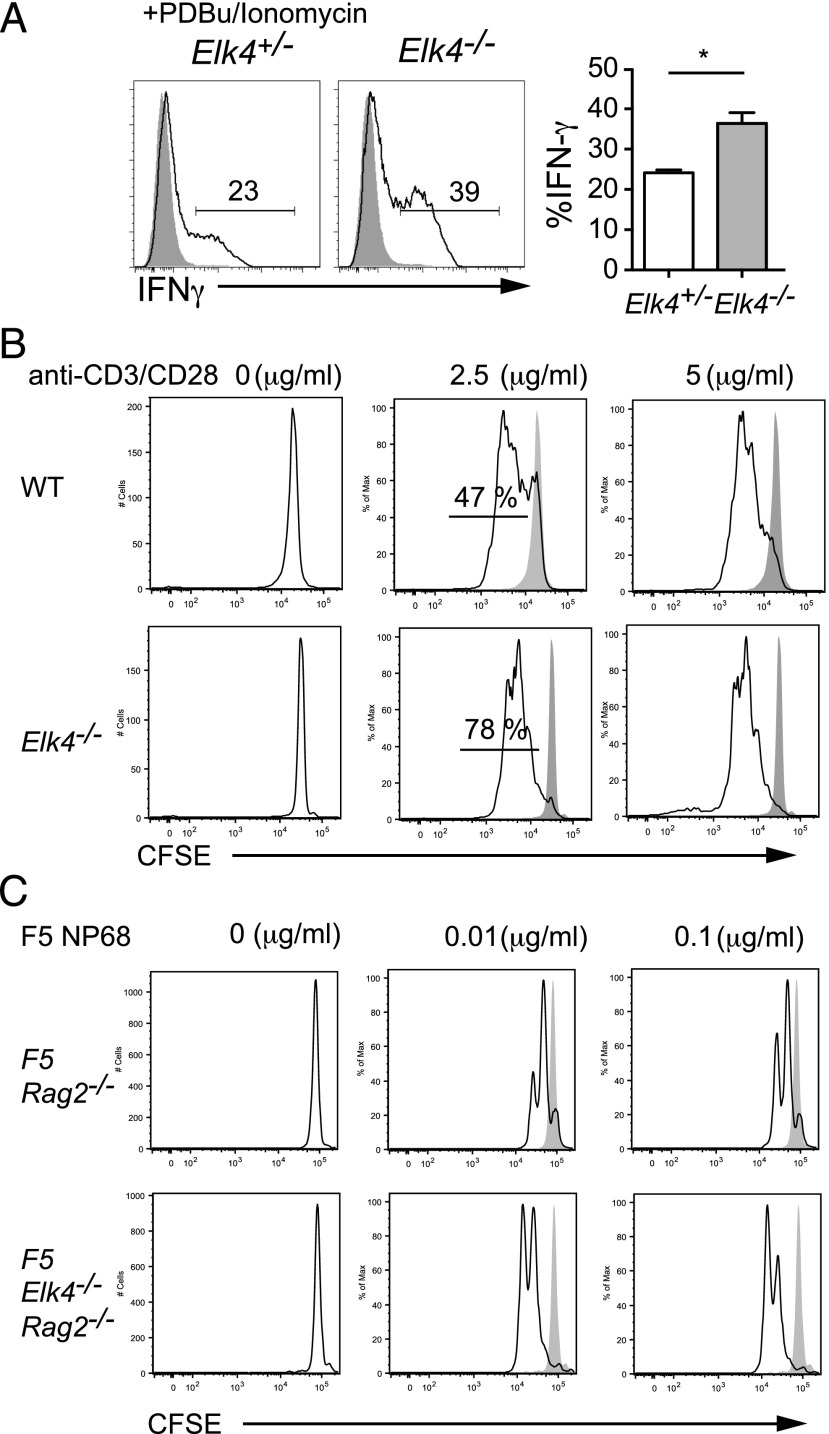
αβ CD8^+^ innate-like T cells have memory-like properties. (**A**) Purified αβ TCRβ^hi^ CD8^+^ SP thymocytes from *Elk4^+/−^*and* Elk4^−/−^* thymus were activated with PDBu and ionomycin and stained for intracellular IFN-γ 5 h later. Three animals per genotype; error bars represent SEM. Statistical significance: *p < 0.05 (unpaired *t* test). (**B**) Proliferation of CFSE-labeled FACS-sorted αβ CD8^+^ SP WT and *Elk4^−/−^* thymocytes stimulated by CD3/CD28 for 48 h. The percentage of divided cells is shown. Two independent experiments were performed. (**C**) Proliferation of CFSE-labeled FACS-sorted *F5 Rag2^−/−^* and *F5 Elk4^−/−^Rag2^−/−^* CD8^+^ thymocytes 48 h following stimulation with NP68 peptide and irradiated splenocytes from C57BL6 (APCs). Two independent experiments were performed.

We assessed the functionality of *Elk4*-null and *Elk4 Elk1*–null αβ CD8^+^ TCRβ^hi^ thymocytes. Upon stimulation with PDBu and ionomycin, purified *Elk4*-null αβ CD8^+^ SP thymocytes produced substantially increased amounts of IFN-γ, a heightened effector function associated with both memory and innate T cells (14, 27, 28) (Fig. 2A). Furthermore, *Elk4*-null αβ CD8^+^ thymic T cells exhibited an enhanced sensitivity to TCR signal, as judged by their proliferative response under limiting activation conditions (Fig. 2B). To exclude the possibility that this reflects altered TCR repertoire, we also examined cells expressing a defined TCR. As with polyclonal αβ CD8^+^ thymic T cells, *Elk4 Rag2*–null F5 transgenic thymic T cells also exhibited enhanced proliferation compared with their WT counterparts upon activation by F5 cognate NP68 peptide (Fig. 2C). Taken together, these observations are consistent with the idea that reduced ELK4–SRF signaling promotes development of αβ CD8^+^ T cells with innate-like characteristics.

### Innate-like CD8^+^ T cells develop in the thymus

**FIGURE 3.**
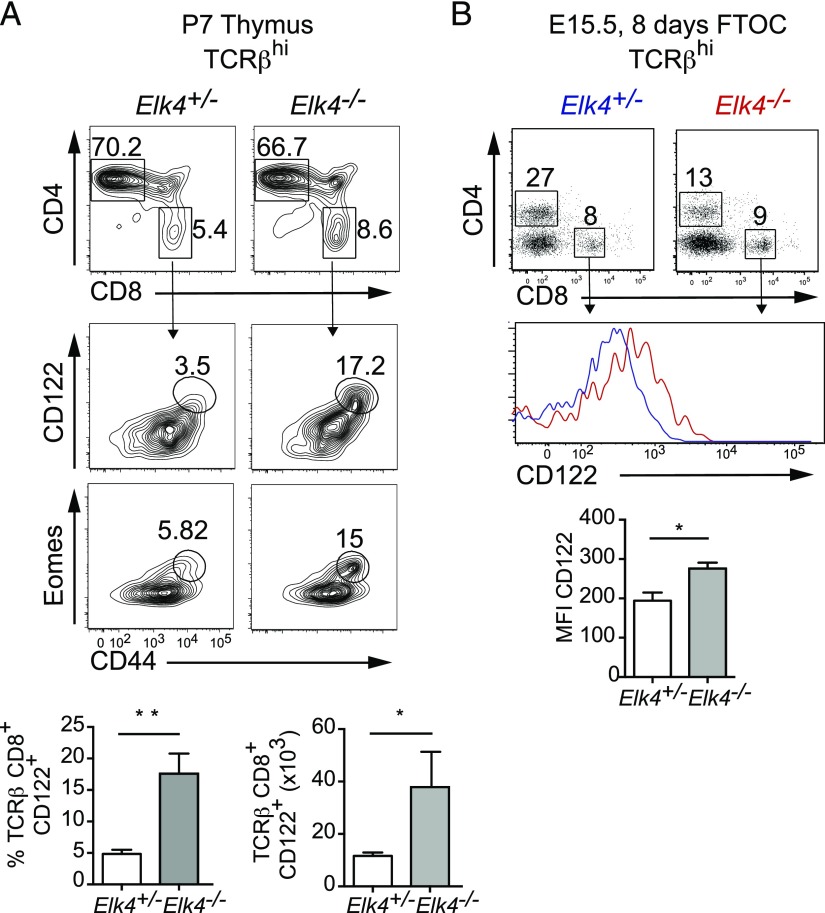
Innate CD8^+^ T cells develop in the thymus. (**A**) Percentages of CD4 and CD8 cells in TCRβ^hi^ thymocytes isolated from P7 *Elk4^+/−^* and *Elk4^−/−^* littermates (top panels) and expression of CD44, CD122, and Eomes in TCRβ^hi^ CD8^+^-gated thymocytes (middle panels). Bottom, Proportions and absolute numbers of TCRβ^hi^ CD122^+^ CD8^+^ innate-like T cells. *n* ≥ 5 animals per genotype. (**B**) Profile of thymocytes stained with anti-CD4 and anti-CD8 from FTOC at day 8 (upper panel). Representative histogram of CD122 expression on TCRβ^hi^ CD8^+^-gated thymocytes from *Elk4^+/−^* (blue) and *Elk4^−/−^* (red) from day 8 FTOC (center panel). *n* ≥ 4 thymic lobes per genotype. Data are expressed as mean ± SEM. Statistical significance: *p < 0.05, **p < 0.01 (unpaired *t* test).

Activated and memory peripheral T cells can traffic to specific locations, including the thymus (30, 31). The presence of innate-like cells in the thymus could reflect the altered homeostasis of peripheral CD8 T cells migrating back into the thymus because αβ CD8^+^ T cells with memory-like phenotypes are present in peripheral lymphoid organs (see below). To exclude this possibility, we analyzed 1-wk-old animals, in which mature T cells have not yet become established in secondary lymphoid organs. Increased numbers of innate-like cells expressing CD44, CD122, and Eomes were also observed in this setting in *Elk4*-null animals (Fig. 3A). Moreover, similar results were observed upon E15.5 *Elk4*–null FTOC (Fig. 3B). Taken together, these results show that *Elk4* inactivation acts in the thymus to promote the generation of innate-like αβ CD8^+^ T cells.

### Differentiation of Elk4-null innate-like CD8^+^ T cells is cell autonomous

**FIGURE 4.**
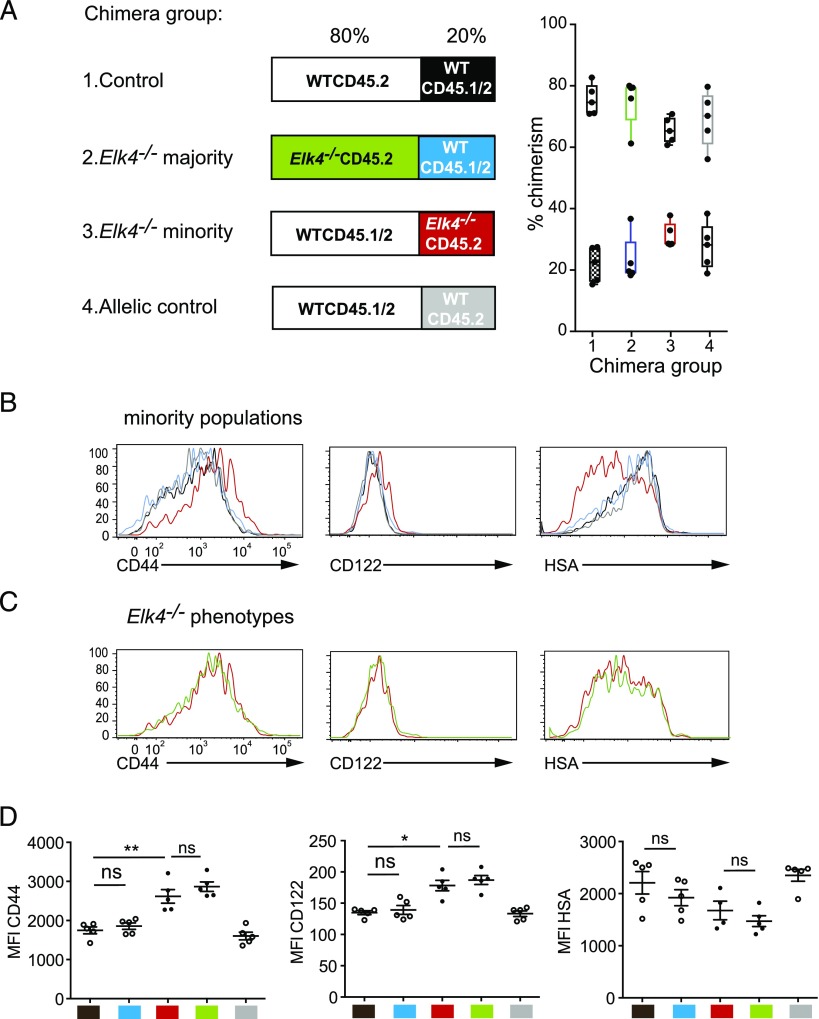
*Elk4^−/−^* αβ CD8^+^ innate-like T cell development in thymus is cell intrinsic. (**A**) Experimental strategy. Four groups of mixed bone marrow were transferred into irradiated CD45.1 hosts. Left panel, Each box indicates the majority and minority genotypes, with details given above, and experimental populations highlighted in color. Group 1: minority WT CD45.1/2 (20%, black), majority WT CD45.2 (80%). Group 2: minority WT CD45.1/2 (20%, blue), majority *Elk4^−/−^* CD45.2 (80%, green). Group 3: minority *Elk4^−/−^* CD45.2 (20%, red), majority WTCD45.1/2 (80%). Group 4: minority WTCD45.2 (20%, gray), majority WT CD45.1/2 (80%). Right panel, Level of chimerism in individual mice analyzed is represented for each group by a box-and-whiskers plot with minimum to maximum value. Each mouse is represented by two data points. (**B**) No bystander effects on minority population. Expression of CD44, CD122, and HSA on the minority TCRβ^hi^ CD8^+^ populations in each group is shown. Black versus blue: no influence of majority *Elk4* genotype on WT cells. Red versus gray: *Elk4^−/−^* phenotype is detectable among majority WT cells. Black versus gray: no influence of CD45 allelic background. (**C**) Expression of CD44, CD122, and HSA on *Elk4^−/−^* cells among WT cells as the minority population (Group 3, red) or the majority population (Group 2, green). (**D**) Summary of results. Mean fluorescence intensity for CD44, CD122, and HSA for each population are color coded as in (A); comparisons made as in (B) and (C). Error bars represent SEM; *n* = 5 animals per group. Similar results were obtained when reconstitution was performed in *Rag2*^−/−^ hosts (Supplemental Fig. 4). Statistical significance: ns, nonsignificant, *p < 0.05, **p < 0.01 (paired *t* test).

Innate-like αβ CD8^+^ T cells can develop either cell autonomously (15–17) or in response to exogenous IL-4 produced by T cell subsets expressing the PLZF transcription factor (12, 13). We used a mixed bone marrow chimera approach to address the issue more directly (Fig. 4A). Irradiated CD45.1 WT host animals were reconstituted with WT bone marrow mixed with marrow from WT or *Elk4*-null animals in either a 4:1 or 1:4 ratio. Six weeks later, the relative contribution and phenotype of the adoptively transferred cells to the thymus population was evaluated, the donor populations being distinguished by their CD45.2 or CD45.1/2 surface markers. WT CD45.1/2 cells exhibited no difference in expression of the CD44, CD122, and HSA memory markers, regardless of whether the original transfer contained excess WT or *Elk4*-null cells (Fig. 4B, 4D, black versus blue). Similarly, *Elk4*-null CD45.2 thymocytes exhibited elevated innate-like marker expression regardless of whether they formed the minority or the majority of the transferred population (Fig. 4C, 4D, red versus green, red versus blue). Similar results were obtained in *Rag2*^−/−^ hosts reconstituted with mixed WT and *Elk4*-null bone marrow (Supplemental Fig. 1).

**FIGURE 5.**
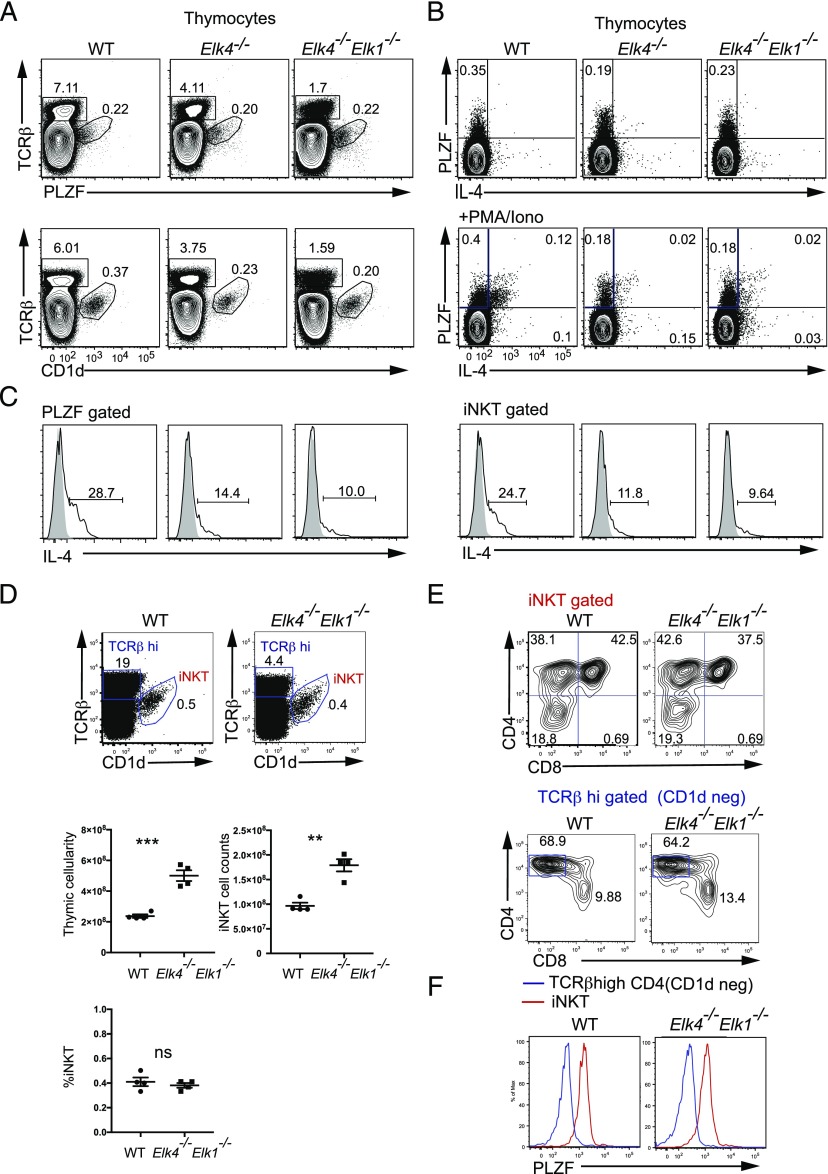
*Elk4^−/−^* αβ CD8^+^ innate-like T cell development does not reflect increased PLZF or IL-4 expression. (**A**). PLZF and IL-4 expression are not increased by *Elk4* and *Elk1* inactivation. Top panels, Analysis of intracellular PLZF, TCRβ, and CD1d expression in WT, *Elk4*^−/−^, and *Elk4*^−/−^ *Elk1*^−/−^ thymocytes (*n* = 7 per genotype). (**B**) Analysis of IL-4 and PLZF expression in unstimulated thymocytes (top panel) and PMA and ionomycin-stimulated thymocytes (bottom panel). (**C**) IL-4 expression in PLZF-gated and CD1d (iNKT)-gated populations (gray solid, unstimulated; black line, +PMA/ionomycin). PLZF-gated: WT, 33.17 ± 3.835%; *Elk4*^−/−^, 15.97 ± 1.927%; *Elk4^−/−^ Elk1^−/−^*, 9.377 ± 0.3699%; *n* = 3, *p* < 0.01. iNKT-gated thymocytes: WT, 26.3 ± 2.335%; *Elk4*^−/−^, 12.9 ± 1.68%; *Elk4^−/−^ Elk-1^−/−^*, 8.527 ± 0.5643%; *n* = 3, *p* < 0.01. (**D**) Proportions and cell numbers of iNKT cells in *Elk4 Elk1*–null thymus. (**E**) Unchanged proportions of CD4 and CD8 cells in iNKT populations and *Elk4^−/−^ Elk1^−/−^* TCRβ^hi^ CD1d^−^ cells. (**F**) PLZF expression levels are unchanged in *Elk4^−/−^ Elk1^−/−^* iNKT and TCRβ^hi^ CD4^+^ CD1d^−^ cells. Data are expressed as mean ± SEM. Statistical significance: **p < 0.01, ***p < 0.001 (unpaired *t* test).

We next investigated PLZF and CD1d expression in *Elk4 Elk1*–null thymus. The proportions of thymocytes expressing PLZF or CD1d were not affected, but the absolute number of iNKT cells was increased in line with the increased thymic cellularity observed in *Elk4 Elk1*–null mutant mice (Fig. 5A, 5D). The intracellular induction levels of IL-4 after PMA/ionomycin stimulation were, however, significantly reduced in *Elk4 Elk1*–null mice, both in total thymocytes (Fig. 5B) and in PLZF-gated and iNKT-gated thymocytes (Fig. 5C). Among individual PLZF-expressing thymocyte populations, the frequency of iNKT cells was unaffected, although their numbers increased, reflecting increased thymic cellularity (Fig. 5D), but the proportion of CD4^+^ TCRβ^hi^ CD1-d tetramer-negative thymocytes was reduced (Fig. 5E). PLZF expression levels in both populations were unaffected (Fig. 5F). Taken together, these data show that generation of *Elk4*-null innate-like αβ CD8^+^ T cells is substantially cell autonomous and does not reflect alterations in PLZF expression and increased IL-4 expression.

### Innate-like αβ CD8^+^ cell development requires immediate-early gene expression

**FIGURE 6.**
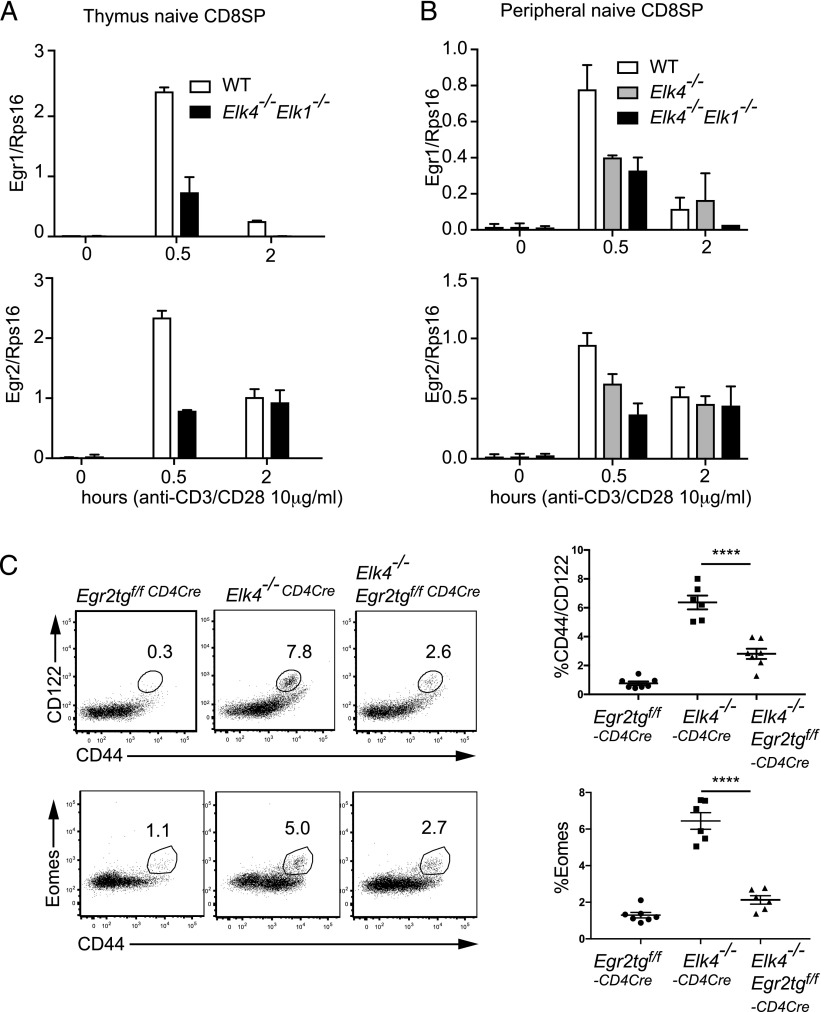
Reduced immediate-early gene expression is associated with innate-like CD8^+^ T cell development. (**A**) CD8 SP TCRβ^hi^ thymocytes and (**B**) peripheral CD8 SP CD44^lo^ CD122^lo^ cells were activated using αCD3 and αCD28, and expression of *Egr1* and *Egr2* at the indicated times was analyzed by quantitative RT-PCR. (**C**) *Rag2*^−/−^ animals were reconstituted either with bone marrow from animals carrying a conditional EGR2 transgene, along with a CD4-Cre transgene to allow expression of EGR2 at the DP stage of thymocyte development, with bone marrow from *Elk4^−/−^ CD4-Cre*, or a combination of these genotypes. TCR β^hi^ CD8 thymocytes were analyzed for CD44, CD122, and intracellular Eomesodermin as in Fig. 1. Error bars represent SEM; *n* ≥ 5 animals per group. Statistical significance: ****p < 0.0001 (unpaired *t* test).

Studies in double-positive (DP) thymocytes have shown that TCR signaling to ELK4 and ELK1 activates the classical immediate-early gene set including Egr and AP1 family members (19, 20). Quantitative RT-PCR confirmed that both in αβ CD8^+^ TCRβ^hi^ thymocytes and peripheral naive αβ CD8^+^ T cells, inactivation of *Elk4* led to reduced transcription of classical immediate-early genes at early times following TCR cross-linking (Fig. 6A, 6B). We previously showed that ectopic expression of Egr family members could rescue the positive selection defect in *Elk4*-null animals (20). To test the role of ELK4–SRF target genes in innate-like T cell development, we used a conditional transgene approach to express EGR2 in DP thymocytes (25). *Egr2* expression partially rescued the positive selection defect in *Elk4*-null animals, reducing the proportion of CD44^hi^ CD122^hi^ CD8^+^ thymocytes and Eomes expression levels (Fig. 6C). These data are consistent with a role for EGR2 and potentially other Egr family members or other ELK4–SRF targets in development of innate-like CD8^+^ T cells in the thymus.

### Innate-like Elk4-null CD8^+^ T cells accumulate in the periphery

**FIGURE 7.**
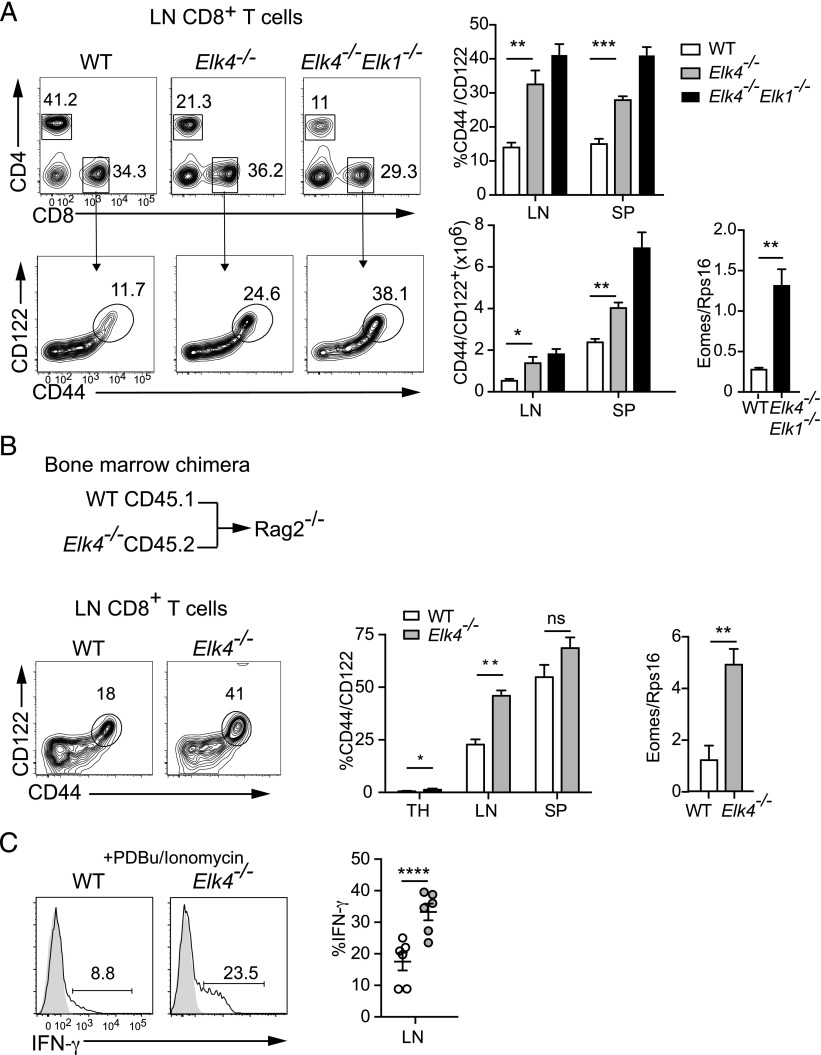
Peripheral αβ CD8^+^ memory-like T cells in *Elk4*-null animals do not arise through lymphopenia-induced proliferation. (**A**) Increased proportions and numbers of CD44^+^ CD122^+^ CD8 T cells in lymph nodes and spleen of primary animals. Levels of Eomes transcripts in purified CD8^+^ lymph node (LN) T cells. (**B**) Analysis of CD8^+^ T cells from *Rag2*^−/−^ hosts reconstituted with WT CD45.1 and *Elk4^−/−^* CD45.2 bone marrow at 1:1 ratio. Increased proportions of CD44^+^ CD122^+^ CD8^+^ SP T cells in thymus (TH; WT, 0.53 ± 0.23%; *Elk4 ^−/−^*, 1.34 ± 0.47%; 10 mice each), LN, and spleen (SP) (*n* ≥ 8 mice). Right, Increased expression of Eomes transcripts in *Elk4 ^−/−^* sorted LN CD8^+^ cells. (**C**) Left, Representative flow cytometry plots of intracellular production of IFN-γ in LN CD8^+^ T cells from animals reconstituted as in (B) after stimulation with PDBu and ionomycin. Right, Data summary (*n* = 6). Data are expressed as mean ± SEM. Statistical significance: *p < 0.05, **p < 0.01, ***p < 0.001, ****p < 0.0001 [unpaired (A) and paired (B and C) Student *t* test].

We next investigated whether *Elk4* inactivation affected accumulation of innate-like T cells in the periphery. Increased numbers of CD44^hi^ CD122^hi^ αβ CD8^+^ T cells, expressing higher levels of Eomes transcripts, were present in the lymph nodes and spleens of *Elk4*- and *Elk4 Elk1*–null animals (Fig. 7A). Similar to αβ CD8^+^ SP thymocytes, these cells produced more IFN-γ after short stimulation and exhibited enhanced sensitivity in response to TCR signaling, as detected by tritiated thymidine incorporation and CFSE staining (Supplemental Fig. 2). *Elk4*-null mice are defective in thymocyte positive selection and exhibit lymphopenia (19), which can promote differentiation of memory-like CD8^+^ T cells in the periphery (14). To exclude this possibility, we analyzed peripheral T cells in animals reconstituted with equal proportions of *Elk4*-null and WT bone marrow. Again, both lymph nodes and spleen in these animals contained elevated proportions of *Elk4*-null CD44^hi^ CD122^hi^ αβ CD8^+^ T cells that expressed Eomes transcripts at a high level (Fig. 7B) and that produced high levels of IFN-γ following stimulation with PDBu and ionomycin (Fig. 7C). Elevated numbers of thymic and peripheral innate-like αβ CD8^+^ T cells were also detected in mixed reconstitutions with *Elk4*-null cells expressing the MHC class I–specific F5 TCR (Supplemental Fig. 3) and so are unlikely to arise because of altered TCR repertoire in *Elk4*-null animals. These results suggest that the increased number of peripheral memory-like αβ CD8^+^ T cells is not a result of lymphopenia in *Elk4*-null animals.

### Reduced ERK signaling in peripheral T cells promotes development of cell with innate-like properties

We next investigated whether memory-like CD8 T cells could be generated de novo in the periphery. Previous work has shown that weak TCR signals in peripheral CD8^+^ T cells, generated by interaction with self-peptide/MHC, promote the differentiation into innate-like cells with memory characteristics (reviewed by Ref. 14). Our results suggest a model in which *Elk4* inactivation effectively decreases ERK signaling to transcription downstream of the TCR, so we tested whether directly decreasing ERK signaling to ELK4 in WT peripheral cells would increase their differentiation into memory-like cells. We used a system in which naive T cells are subjected to TCR cross-linking in vitro, followed by culture in IL-2; this treatment induces T bet and Eomes expression and causes them to produce IFN-γ and TNF immediately upon subsequent TCR restimulation (32). F5 *Rag2*–null CD8^+^ T cells were used to study the response of cells expressing a defined TCR.

**FIGURE 8.**
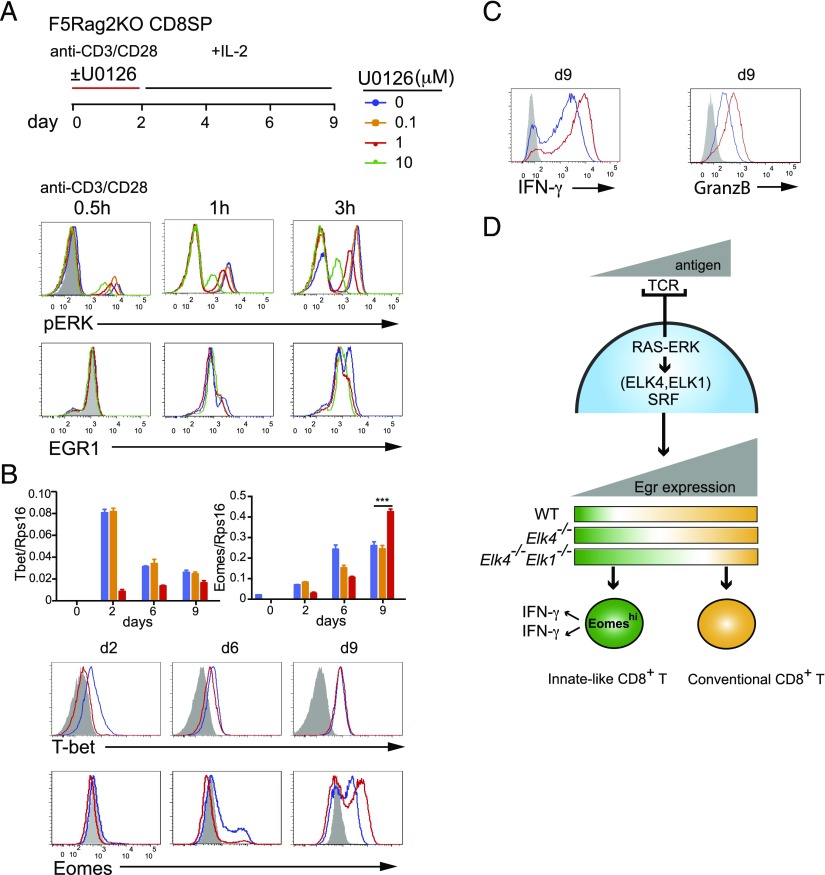
Inhibition of ERK signaling promotes differentiation of naive peripheral αβ CD8^+^ T cells into cells with memory-like characteristics. (**A**) Differentiation of naive WT F5 *Rag2*^−/−^ CD8^+^ T cells. Peripheral CD8^+^ T cells were activated for 2 d with anti-CD3/anti-CD28 (10 μg/ml), with or without U0126 addition, and then cultured in IL-2 as indicated. Intracellular activated phospho-ERK (top) or Egr-1 protein (bottom) following anti-CD3 and anti-CD28 activation. Gray shading, isotype control; color-coded lines, WT F5 *Rag2*^−/−^ cells according to U0126 concentration. (**B**) Expression of Eomes and T-bet. Top, Quantitative RT-PCR analysis. Bottom, Flow cytometric analysis of protein levels. (**C**) Flow cytometric analysis of intracellular IFN-γ and granzyme B expression after PDBu/ionomycin activation for 5 h. Data are representative of six (A–C) independent experiments, each done in triplicate. Data are mean ± SEM. Statistical significance: ***p < 0.001 (unpaired *t* test). (**D**) Summary of the relationship between MHC-peptide–TCR avidity, activation of TCF-SRF target genes, and cell differentiation pathways in thymus and periphery. Inactivation of ERK-ELK4/ELK1–SRF signaling in thymocytes and in peripheral αβ CD8^+^ T cells favors the development of cells with innate-like characteristics, which exhibit enhanced Eomes expression and immediate production of IFN-γ.

Inclusion of the MEK inhibitor U0126 during the initial TCR ligation phase progressively limited both ERK activation and *Egr1* expression (Fig. 8A). Submaximal concentrations of U0126 reduced T-bet expression at early times of IL-2 culture and increased Eomes expression at late times (Fig. 8B). After prolonged culture in IL-2, U0126-treated F5 αβ CD8^+^ T cells exhibited increased production of IFN-γ and granzyme B upon stimulation with PDBu and ionomycin (Fig. 8C). Similar results were obtained when we examined purified naive CD44^lo^ *Elk4*-null αβ CD8^+^ T cells in this assay (Supplemental Fig. 4A). Experiments with T cells expressing the F5 TCR showed that these changes were also dependent on SRF, the targeting partner protein for ELK4 (data not shown). Together, these data are consistent with a model in which decreased ERK signaling to ELK4 target genes in peripheral or thymic αβ CD8^+^ T cells favors the development of T cells with memory-like characteristics (Fig. 8D).

## Discussion

TCR signaling to transcription via the Ets protein ELK4 (SAP-1), its relative ELK1, and their partner transcription factor SRF is critical for thymocyte positive selection (19–22). In this study, we have shown that ELK4 and ELK1 act redundantly and cell autonomously downstream of ERK signaling to limit the generation of innate-like αβ CD8^+^ T cells with memory characteristics in the thymus. These cells exhibit hallmarks of conventional memory CD8^+^ T cells, with enhanced expression of the T-box transcription factors Eomes and T-bet and increased proliferation and production of IFN-γ in response to TCR ligation (27–29). The effect of ELK4 inactivation is unlikely to reflect a change in TCR repertoire that preferentially selects innate-like cells because increased innate-like αβ CD8^+^ differentiation occurred in *Elk4*-null cells expressing the MHC class I–specific F5 TCR. Moreover, innate-like cells were also present in neonatal and embryonic animals, in which the TCR repertoire is limited (33). Like other ELK4 phenotypes, generation of innate-like CD8+ T cells was further enhanced by additional inactivation of *Elk1*. Transgenic expression of the ELK4 target gene *Egr2* reduced the innate-like αβ CD8^+^ T cell development in *Elk4*-null animals and rescued the positive selection defect. Our results are consistent with the proposal that innate-like differentiation is favored by low TCR signal strength (6, 7) (Fig. 8D).

We used a chimeric reconstitution approach to show that ELK4 acts cell autonomously in innate-like αβ CD8^+^ T cell formation and that this is associated with increased Eomes expression. Innate-like αβ CD8^+^ T cell formation is also controlled cell autonomously by *Nfkb1* (16, 34) and *Bcl11b* (15), and *Irf4* acts partly cell autonomously (17, 35). Previous work has demonstrated that generation of innate-like αβ CD8^+^ T cells in the thymus and periphery can also be induced by cell-extrinsic pathways. Genes, such as *Itk*, *Klf2*, *Id3*, and *Cbp*, influence innate-like T cell development by increasing levels of IL-4, produced by elevated numbers of PLZF^+^ cells such as iNKT and CD4^+^ TCRβ^hi^ cells (12, 13, reviewed by Ref. 14). However, we found that proportions of iNKT cells were unchanged in *Elk4 Elk1*–null thymocytes, whereas CD4^+^ TCRβ^hi^ cells were decreased, and PZLF expression levels were unchanged in both cell subsets. Moreover, innate-like thymic αβ CD8^+^ T cells in *Elk4*-null animals expressed markers associated with conventional or lymphopenia-induced memory cells but not IL4-induced memory. IL-4 production in total PLZF^+^ and iNKT^+^ thymocytes was reduced in *Elk4* null animals and further reduced in *Elk4 Elk1*–null thymocytes. Thus, IL4 signaling cannot account for the increased innate-like αβ CD8^+^ thymocyte differentiation that we observe in Elk4-null animals.

Several studies suggest that in addition to promoting positive selection, TCR signaling controls development of innate-like αβ CD8^+^ T cells (reviewed by Ref. 6, 7). Both *Itk*-null (8, 9) and mutant *Slp76^Y145F^* (10, 11) thymocytes exhibit impaired positive selection and increased innate αβ CD8^+^ T cell numbers. Moreover, these phenotypes can be partially suppressed by expression of the hypersensitive ERK^sem^ protein, suggesting they are directly controlled by ERK signaling (8, 9). Consistent with this idea, we found that inhibition of ERK signaling promoted the differentiation of cultured peripheral T cells to a memory-like phenotype upon TCR ligation and culture in IL2.

The genomic targets of ELK4–SRF signaling include members of the AP-1 and Egr transcription factor families. We found that transgenic expression of EGR2 suppressed development of innate-like αβ CD8^+^ T cells in Elk4-null thymus, suggesting that EGR2 or other Egr family members act downstream of ELK4; however, it is likely that other ELK4–SRF target genes may be involved because the suppression observed was incomplete. Interestingly, inactivation of Id3, an ERK-responsive Egr target gene (36), also increases innate-like αβ CD8^+^ T cell development but does not do so cell autonomously (12). Moreover, unlike ELK4, Id3 inactivation impairs both positive and negative selection (26). Together, these observations indicate that loss of Id3 expression downstream of ELK4 cannot be responsible for innate αβ CD8^+^ T cell development in *Elk4*-null animals.

Our data show that ELK4, a major nuclear ERK target in thymocytes, acts downstream of ERK signaling to suppress innate-like αβ CD8^+^ T cell development, acting via target genes such as *Egr2*. We propose that inactivation of *Elk4* attenuates TCR signaling, thereby effectively mimicking low-affinity TCR signaling, leading to increased numbers of innate-like αβ CD8^+^ T cells in addition to impairing positive selection. We note that ITK signaling acts upstream of SRF (37) and that inhibition of ITK signaling potentiates the effect of *Irf4* inactivation on innate-like αβ CD8^+^ T cell development (17). This suggests that the ELK4–SRF and IRF4 pathways might cooperate, and, indeed, Irf4 protein levels, but not transcripts, are reduced in *Elk4*-null thymus (D. Maurice, unpublished observation). It is interesting to note that AP1 [whose components, such as *JunB,* are *Elk4* target genes in T cells (20)] functionally cooperates with IRF4 (38, 39).

## Supplementary Material

Data Supplement
