# Supplementary material for: ERK Signaling Controls Innate-like CD8+ T Cell Differentiation via the ELK4 (SAP-1) and ELK1 Transcription Factors
Source: J Immunol. 2018 Aug 1;201(6):1681–91. doi: 10.4049/jimmunol.1800704 (PMC6121213; doi:10.4049/jimmunol.1800704)
Supplement: Data Supplement [file JI_1800704.zip › JI_1800704_Supplemental_Figures_1.pdf]

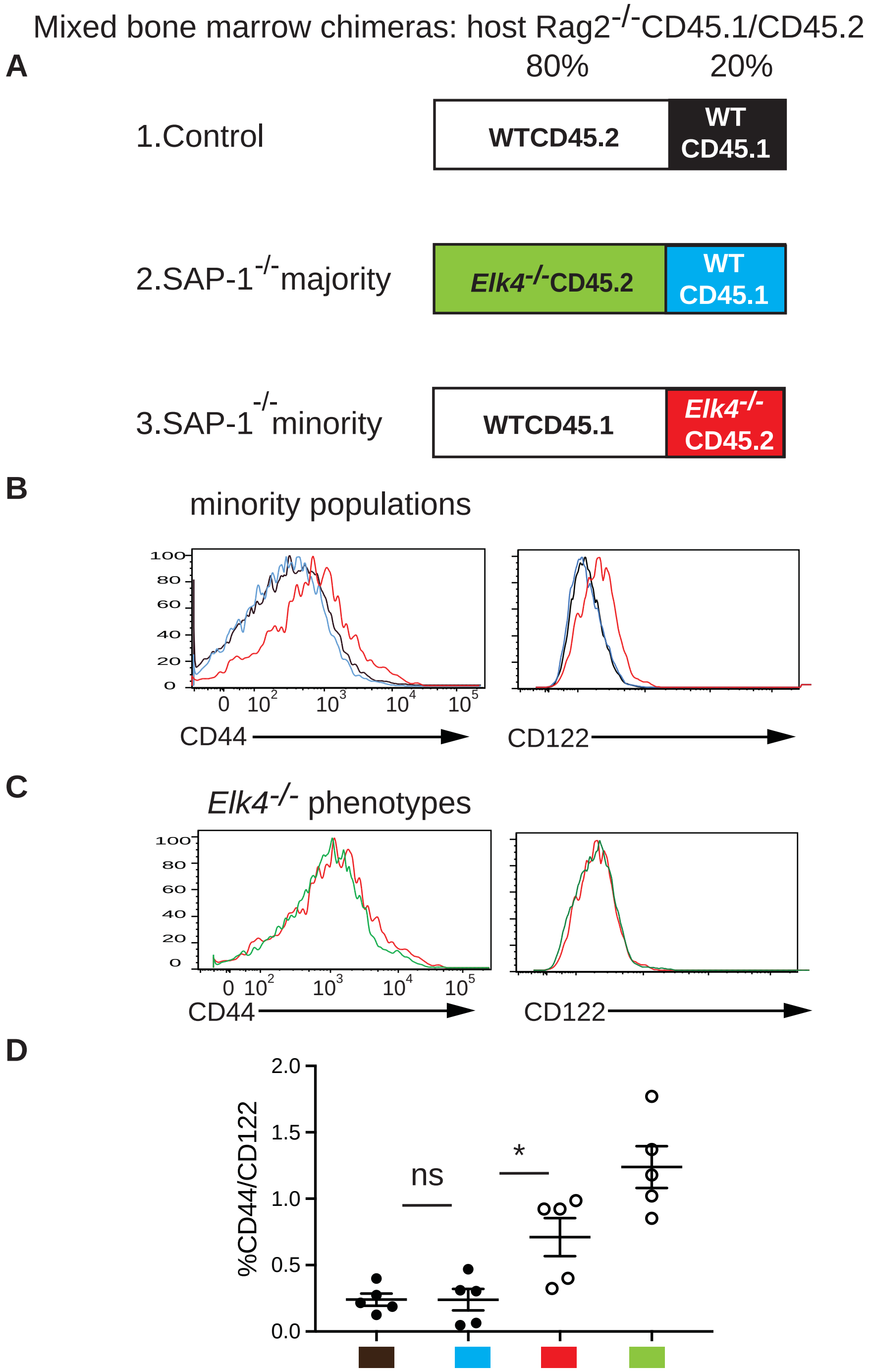

Figure S1. *Elk4*$^{-/-}$ αβ CD8^{+} innate-like T cell development in the thymus is cell intrinsic (A). Experimental strategy: CD45.1/CD45.2 Rag2$^{-/-}$ hosts were reconstituted with WT CD45.1 and *Elk4*$^{-/-}$ CD45.2 bone marrow cells, as in Figure 4 **(B).** CD44 and CD122 profiles of minority populations *Elk4*$^{-/-}$ (red), WT CD45.1 (black and blue) in each bone marrow mix is shown. **(C).** Increased CD44 and CD122 expression profiles on *Elk4*$^{-/-}$ thymocytes regardless of whether they are the majority (green) or the minority population (red). **(D).** Percentage of WT and *Elk4*$^{-/-}$ CD8^{+} SP T cells staining positive for both CD44 and CD122 in each group (n=5 mice). Error bars represent SEM, n ≥ 5 animals per group. Significance: *, p<0.05; **, p<0.01; ***, p<0.001 (paired *t* test).

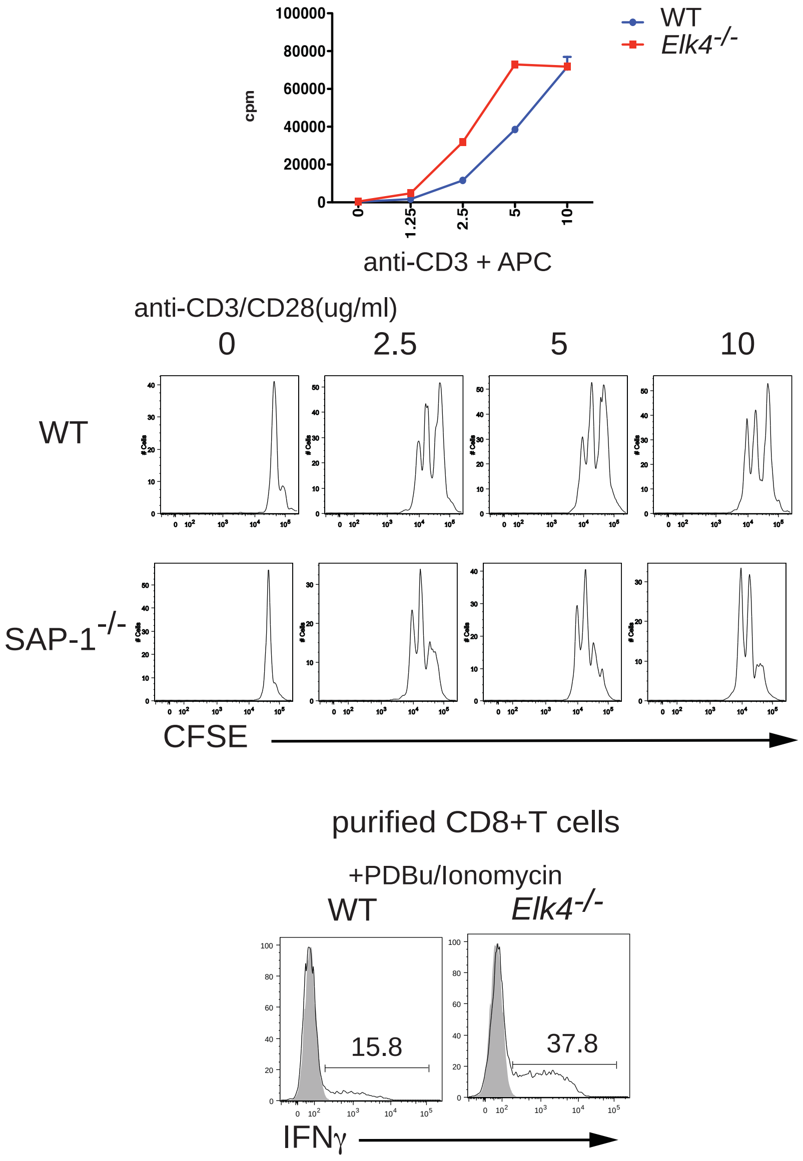

Figure S2. Peripheral CD8$^+$ T cells. Purified peripheral CD8$^+$ LN T cells from WT and *Elk4*$^{-/-}$ animals was assessed for tritiated thymidine incorporation for 48 hours (top) and CFSE dilution (middle) and IFN-γ production (bottom) following 5 hours of PDBu/Ionomycin stimulation All experiments have been performed on at least 5 mice per genotype.

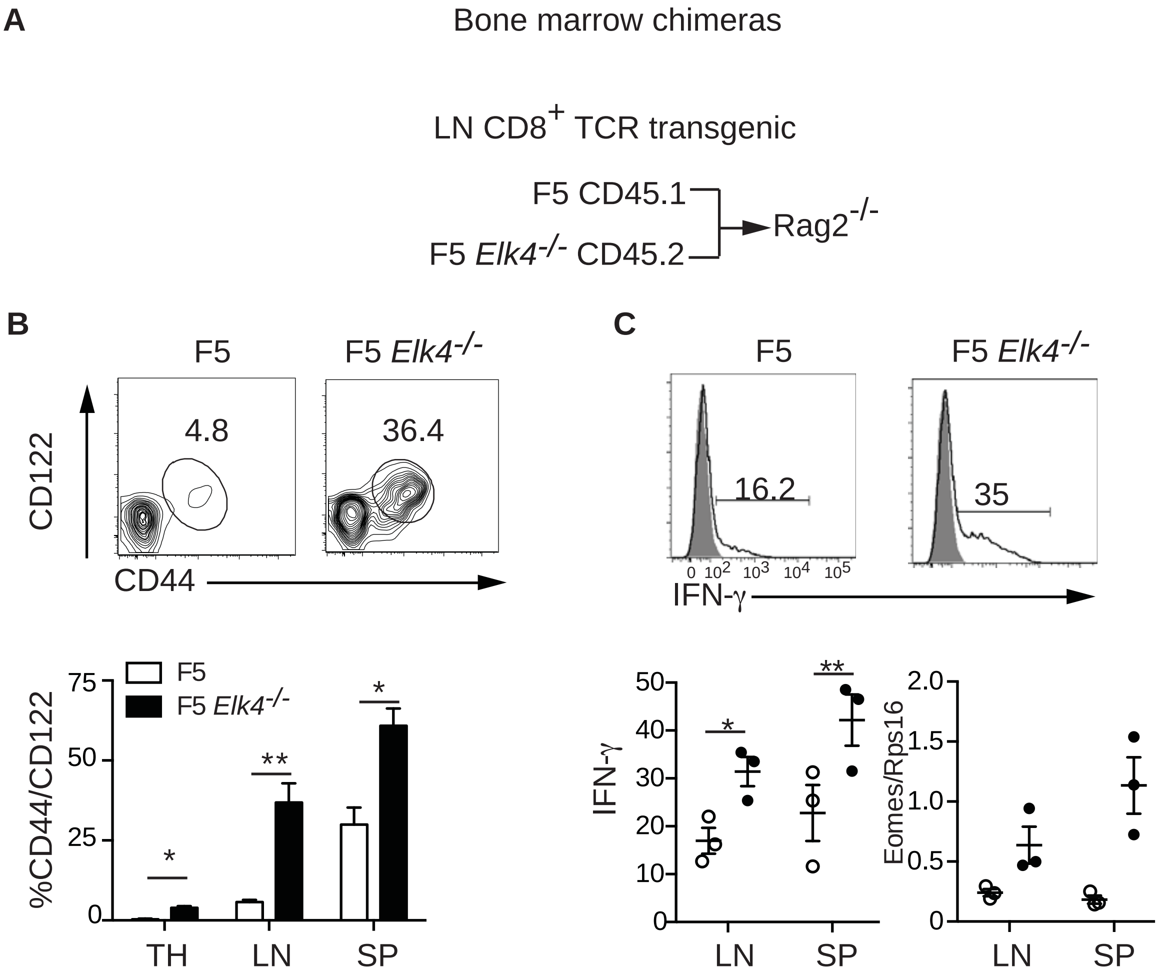

Figure S3. Increased innate-like αβ CD8^{+} T cells numbers in *Elk4*$^{-/-}$ null animals do not reflect changes in TCR repertoire. (A). Transgenic F5 CD45.1 and F5 ***Elk4***$^{-/-}$ CD45.2 bone marrow was used to reconstitute Rag-2$^{-/-}$ hosts at 1:1 ratio. **(B).** Top, representative flow-cytometry plots of CD122 and CD44 expression in F5-TCR transgenic CD8^{+} LN T cells in reconstituted animals. Below, CD44^{+} CD122^{+} CD8^{+} T cells in thymus (TH; WT, 0.3016 ± 0.2291; SAP-1$^{-/-}$, 3.913 ± 0.5140; 3 mice each), lymph nodes (LN) and spleen, n≥ 3 mice. **(C).** Intracellular production of IFN-γ in F5 transgenic T cells after stimulation with PDBu and ionomycin. Below quantification and qPCR analysis of Eomes transcripts in purified LN and SP αβ CD8^{+} T cells. Data are expressed as mean ± SEM. Each symbol represents an individual mouse. Statistical significance: paired Student's t test. *, p <0.05; **, p<0.01; ***, p<0.001; ****, p<0.0001.

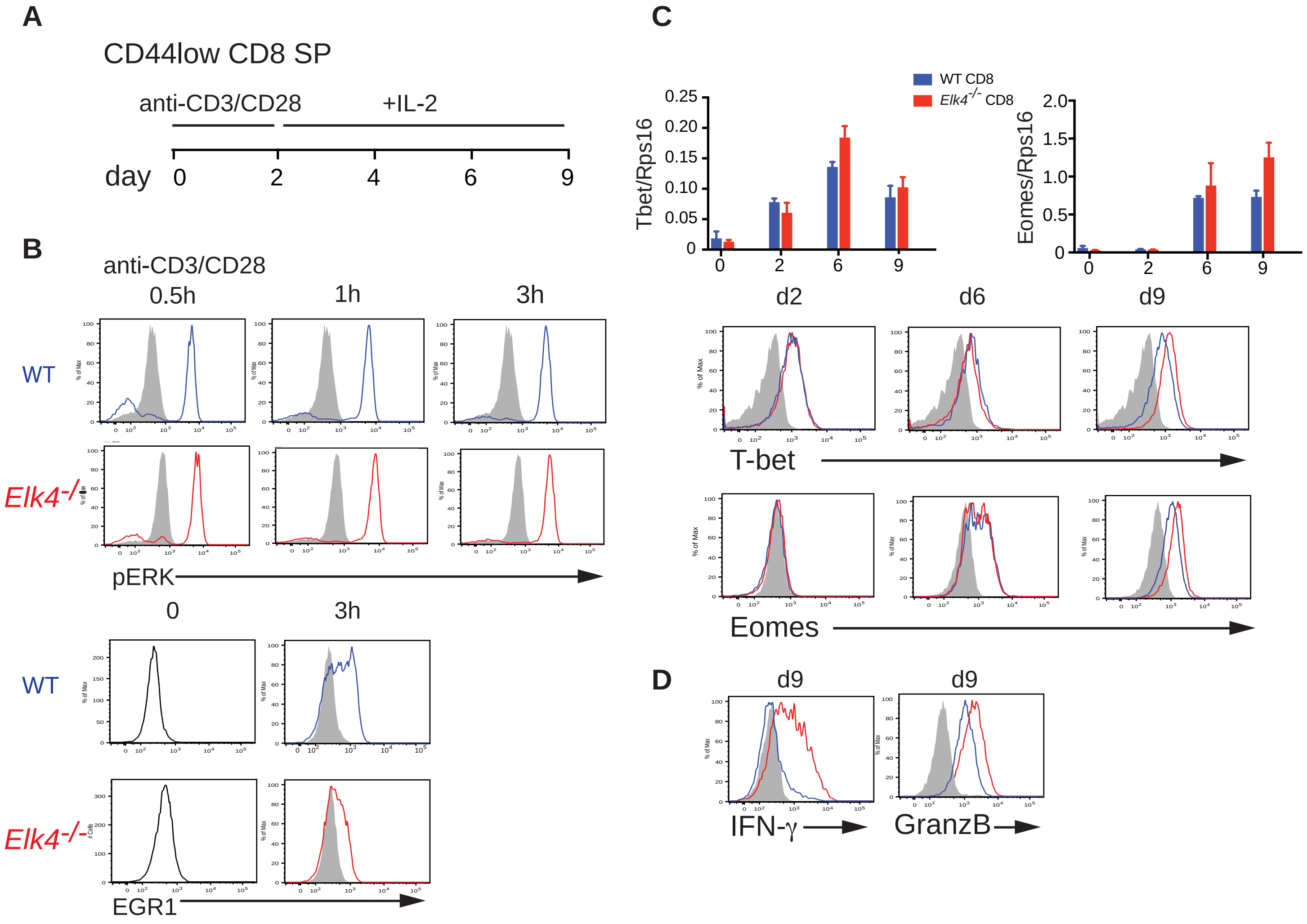

Figure S4. *Elk4*$^{-/-}$ αβ CD8^{+} T peripheral naïve T cells acquire memory-like characteristics. **(A)** Differentiation protocol for sorted SAP-1$^{-/-}$ naïve CD44 low CD8 SP *in vitro* culture. **(B).** ERK signalling was unaffected in *Elk4*$^{-/-}$ null cells, but *Egr1* induction was impaired. **(C)**. No change in T-bet transcription at early times of IL-2 culture, but enhanced Eomes expression at late times. **(C).** Following 9d IL-2 culture, *Elk4*$^{-/-}$ null cells produced substantially increased amounts of IFN-γ and granzyme B 5h after stimulation with PDBu and ionomycin. Experiment has been performed twice.
